# Polyploid giant cancer cells, cytokines and cytomegalovirus in breast cancer progression

**DOI:** 10.1186/s12935-023-02971-1

**Published:** 2023-06-20

**Authors:** Sandy Haidar Ahmad, Ranim El Baba, Georges Herbein

**Affiliations:** 1 Department Pathogens and Inflammation-EPILAB, EA4266, University of France-Comté, 16 Route de Gray, 25030 Besançon Cedex, France; 2 Department of Virology, CHRU Besancon, Besancon, France

**Keywords:** Human cytomegalovirus, High-risk HCMV strains, CTH cells, PGCCs, Cytokines, Breast cancer biopsies

## Abstract

**Background:**

Breast cancer is the most common cancer among women. Accumulated evidence over the past decades indicates a very high prevalence of human cytomegalovirus (HCMV) in breast cancer. High-risk HCMV strains possess a direct oncogenic effect displayed by cellular stress, polyploid giant cancer cells (PGCCs) generation, stemness, and epithelial-to-mesenchymal transition (EMT) leading to cancer of aggressive phenotype. Breast cancer development and progression have been regulated by several cytokines where the latter can promote cancer cell survival, help in tumor immune evasion, and initiate the EMT process, thereby resulting in invasion, angiogenesis, and breast cancer metastasis. In the present study, we screened cytokines expression in cytomegalovirus-transformed HMECs (CTH cells) cultures infected with HCMV high-risk strains namely, HCMV-DB and BL, as well as breast cancer biopsies, and analyzed the association between cytokines production, PGCCs count, and HCMV presence in vitro and in vivo.

**Methods:**

In CTH cultures and breast cancer biopsies, HCMV load was quantified by real-time qPCR. PGCCs count in CTH cultures and breast cancer biopsies was identified based on cell morphology and hematoxylin and eosin staining, respectively. CTH supernatants were evaluated for the production of TGF-β, IL-6, IL1-β, and IL-10 by ELISA assays. The above-mentioned cytokines expression was assessed in breast cancer biopsies using reverse transcription-qPCR. The correlation analyses were performed using Pearson correlation test.

**Results:**

The revealed PGCCs/cytokine profile in our in vitro CTH model matched that of the breast cancer biopsies, in vivo. Pronounced cytokine expression and PGCCs count were detected in particularly CTH-DB cultures and basal-like breast cancer biopsies.

**Conclusions:**

The analysis of cytokine profiles in PGCCs present mostly in basal-like breast cancer biopsies and derived from CTH cells chronically infected with the high-risk HCMV strains might have the potential to provide novel therapies such as cytokine-based immunotherapy which is a promising field in cancer treatments.

**Supplementary Information:**

The online version contains supplementary material available at 10.1186/s12935-023-02971-1.

## Introduction

Polyploid giant cancer cells (PGCCs) play an important role in tumor heterogeneity; they are implicated in tumor initiation, progression, metastasis, and therapy resistance in breast, ovarian, and prostate cancers [1, 2]. PGCCs count was associated with tumor grade and metastasis degree in patients with breast cancer (BC) [3]. PGCCs promote BC metastasis and chemoresistance by modulating the tumor microenvironment (TME) [1, 4]. The presence of polyploidy was identified as a common characteristic among all the tumors initiated by oncoviruses [5]. Human cytomegalovirus (HCMV), a ubiquitous beta-herpesvirus, exhibits a broad cellular tropism [6]. HCMV genome and/or antigens were detected in numerous malignancies including breast, ovarian, prostate, and colon cancer, as well as in neural-derived cancers such as glioblastoma, neuroblastoma, and medulloblastoma. The potential relation between HCMV and cancer was explained by the oncomodulation paradigm [7–12]. Besides the oncomodulation paradigm, our research group highlighted a direct oncogenic effect of high-risk HCMV strains [13–17]. Following human mammary epithelial cells (HMECs) infection, high-risk strains namely, HCMV-DB and HCMV-BL transformed HMECs into cytomegalovirus-transformed HMECs (CTH cells) [16, 17]. PGCCs appearance was described in CTH cultures and associated with enhanced cell proliferation, activation of epithelial-to-mesenchymal transition (EMT), and stemness processes [17, 18]. Moreover, the expression of HCMV-IE1 was identified in PGCCs-CTH cells [17, 18]. These studies highlighted the emergence of PGCCs as a critical factor following HCMV infection.

Numerous studies have described the capacity of cytokines to regulate the induction and progression of breast cancer. Several interleukins including IL-1, IL-6, IL-11, IL-19, and transforming-growth factor β (TGF-β) promoted breast cancer cell proliferation and/or invasion [19, 20]. TGF-β, the most studied cytokine in breast cancer, plays a dual role in tumor progression. At an early stage of tumorigenesis, TGF-β acts as a tumor suppressor due to its anti-proliferative effects. At late stages, TGF-β induces tumor progression by enhancing cancer cell invasion, survival, and immune evasion [21–24]. Furthermore, the presence of TGF-β in the microenvironment correlates with tumor progression and poor prognosis [22]. IL-6 is capable to convert non-stem cancer cells to cancer stem-like cells in breast and prostate cell lines [25]. IL-1 and IL-10 are highly expressed in high grade breast cancer [26–28]. Further, the IL-1 was associated with cancer cells’ proliferation, invasion, angiogenesis, and breast cancer metastasis [29, 30]. IL-6, and IL-1 activate NF-kB and increase cyclin D1 in the normal breast cells causing a neoplastic phenotype [31]. Furthermore, IL-6 and TGF-β initiate the epithelial-to-mesenchymal transition (EMT) process [24, 32–35] leading to cancer progression and metastasis [34, 36, 37]. Additionally, some cytokines including TGF-β, IL-10 and IL-6 were described to facilitate tumor escape [38–41]. Herein, we screened cytokines expression in CTH cultures as well as breast cancer biopsies, and analyzed the link between cytokines production, PGCCs, and HCMV presence in vitro and in vivo.

## Materials and methods

### Cells cultures

Human primary mammary cells (HMECs) were purchased from Life Technologies (Carlsbad, CA, USA). HMECs were cultured in HMEC medium supplemented with HMEC supplement, bovine pituitary extract, and penicillin/streptomycin (Life Technologies) at 37 °C, 5% CO2, and 95% humidity. HMECs were infected with high-risk strains HCMV-DB (KT959235) and HCMV-BL (MW980585) at MOI of 1 as previously described [17, 42]. Following chronic infection, CMV-Transformed HMECs (CTH) cells were emerging and cultured in the same conditions as HMECs. CTH cells were maintained in culture for more than 12 months. Mycoplasma contamination status was monitored on a monthly basis for all cultures (VenorGem classic mycoplasma detection, Minerva biolabs).

### Breast biopsies

Healthy breast biopsies (n = 4) and breast cancer biopsies (n = 16: luminal tumor biopsies n = 8 and basal tumor biopsies n = 8) were provided by the Regional Tumor Bank (BB0033-00024 Tumorothéque Régionale de Franche-Comté). The local ethics committees of Besançon University Hospital (Besançon, France) and the French Research Ministry (AC-2015-2496, CNIL n°1173545, NF-S-96900 n ° F2015) permitted the study. All patients provide their written informed consent to participate in the study.

### Viral detection

Real-time qPCR was used to assess the presence of HCMV in CTH cultures and breast cancer biopsies. DNA was extracted from CTH supernatants using E.Z.N.A. Blood DNA Kit, D3392-02, Omega BIO-TEK). Genomic DNA isolated from patient breast tumor biopsies and healthy human breast tissue was provided by the Regional Tumor Bank (BB0033-00024 Tumorothèque Régionale de Franche-Comté). Viral load was quantified by qPCR using KAPA SYBR FAST Master Mix (KAPA BIOSYSTEMS, KK4601) and IE1 primers (Forward 5'-CGACGTTCCTGCAGACTATG-3' and reverse 5'-TCCTCGGTCACTTGTTCAAA-3') according to the manufacturer’s protocol. Reactions were activated at 95 °C for 10 min, followed by 50 cycles (15 s at 95 °C and 1 min at 60 °C). Real-time qPCR reactions were conducted using a Stratagene Mx3005P thermocycler (Agilent). Results were analyzed using MxPro qPCR software.

### PGCCs detection and count in CTH cultures and biopsies

CTH cells were monitored by an Olympus optical microscope (Japan) and OPTIKA microscopy digital camera (Opticam, Italy). PGCCs present in CTH cultures were identified and counted based on cell morphology as previously reported [18]. PGCCs presence in BC biopsies was confirmed by hematoxylin and eosin staining based on Zhang et al. PGCCs description [43]. PGCCs quantification was similarly performed for all breast cancer biopsies. Briefly, PGCCs were counted in five hot spots of each tumor sample in hematoxylin and eosin slides (magnification 400X, field diameter 0.45 mm).

### Cytokines production and expression in CTH cultures and biopsies

Supernatants from CTH cultures were harvested on different days post-infection and evaluated for the presence of cytokines. ELISA kits were used for the detection of Human TGF-β (kit reference 650.010.096, Diaclone, France), human IL-6 (kit reference 851.520.001, Diaclone, France), human IL-1β (kit reference 851.610.001, Diaclone, France), and human IL-10 (kit reference 851.540.005, Diaclone, France) in CTH supernatants. ELISA assays were performed according to the manufacturer’s protocol.

Cytokines expression in breast cancer biopsies was evaluated by reverse transcription-quantitative polymerase chain reaction (RT-qPCR). Total RNA was extracted from BC biopsies using E.Z.N.A. Total RNA Kit I (Omega Bio-Tech, GA, USA). Following DNase I treatment (ThermoFisher), reverse transcription was performed using the SuperScript IV First-Strand Synthesis kit (Invitrogen, Carlsbad, CA, USA). Expression of TGF-β, IL-6, IL1-β, and IL-10 was measured by real-time qPCR using KAPA SYBR FAST Master Mix (KAPA BIOSYSTEMS, KK4601) and specific primers (listed in Additional file 1: Table S1) according to the manufacturer’s protocol. The cytokines analyzed in the tissue samples were normalized to the housekeeping gene GAPDH. The GAPDH primers used are listed in Additional file 1: Table S1.

### Statistics

The statistic software SPSS 23 was used to analyze the data. Correlation analyses were performed using Pearson correlation test. p-value ≤ 0.05 was considered significant. Plots and histograms were executed using Microsoft Excel. Data are presented as mean ± SD of two independent experiments.

## Results 

### Kinetics of PGCCs appearance in CTH cultures

**Fig. 1:**
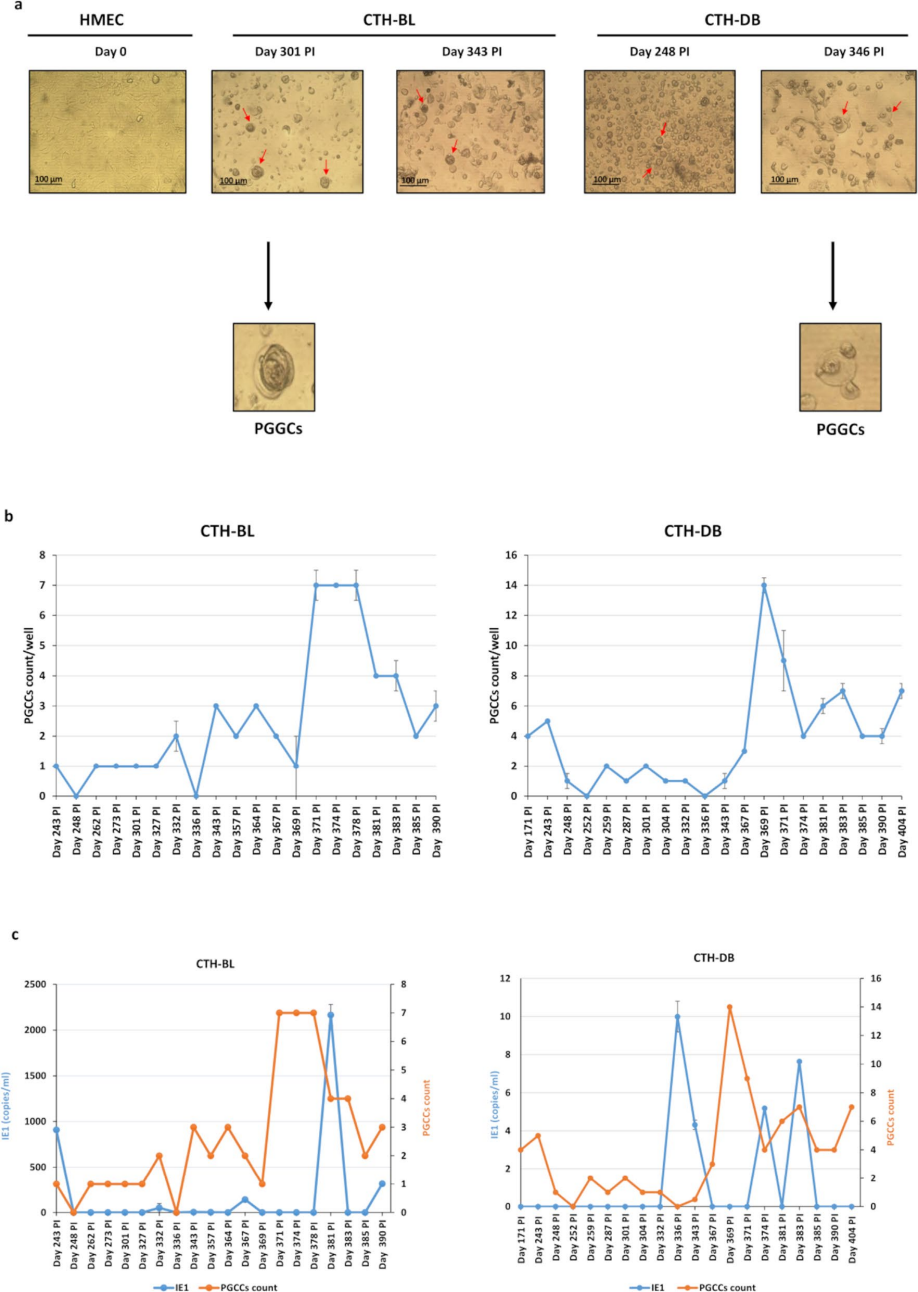
PGCCs detection in CTH cultures. **a** An inverted light microscope was used to monitor the chronic CMV-transformed-HMECs (CTH)-BL and CTH-DB cultures, Magnification × 100, scale bar 100 μm. **b** Curves representing the PGCCs count detected in CTH cultures. The Y-axis represents the PGCCs count /well, and the X-axis represents the days post-infection. **c.** Time-course of the viral load in the CTH-DB and BL culture as measured by IE1-qPCR. Data are represented as mean ± SD of two independent experiments. Blue and orange curves represent the viral load (copies/ml) as measured by IE1-qPCR (Left Y-axis) and PGCCs count/well (Right Y-axis), respectively. The X-axis represents the days post-infection

CTH cells were maintained in culture for more than 12 months as previously reported [17, 18]. After chronic infection, CTH cells exhibit an extremely heterogeneous population. Compared to uninfected HMECs, we identified the presence of large cells with morphological heterogeneity in chronic CMV-transformed-HMECs (CTH)-BL and CTH-DB (Fig. 1a, red arrows). Besides the presence of small-sized cells, giant cells with large nuclei, as well as giant cells with blastomere-like morphology and mesenchymal cells, were identified in CTH cultures parallel to the asymmetric cell division patterns; these giant cells were named PGCCs as previously reported [17, 18]. The PGCCs count was monitored from day 171 to 404 and from day 243 to 390 in chronically infected CTH-DB and CTH-BL cultures, respectively. The PGCCs count in CTH-BL cultures increased slightly at day 343 post-infection and reached a peak at days 371, 374, and 378 post-infection. A higher PGCCs count was observed in CTH-DB cultures, especially at day 369 post-infection (Fig. 1b). Furthermore, we assessed the presence of HCMV in CTH cultures using real-time qPCR. HCMV viral load was higher in CTH-BL compared to CTH-DB cultures. Viral replication was noticed in the presence of low PGCCs count in CTH cultures (Fig. 1c).

### Kinetics of cytokines production (TGF-β, IL-1β, IL-6, and IL-10) in CTH cultures

**Fig. 2:**
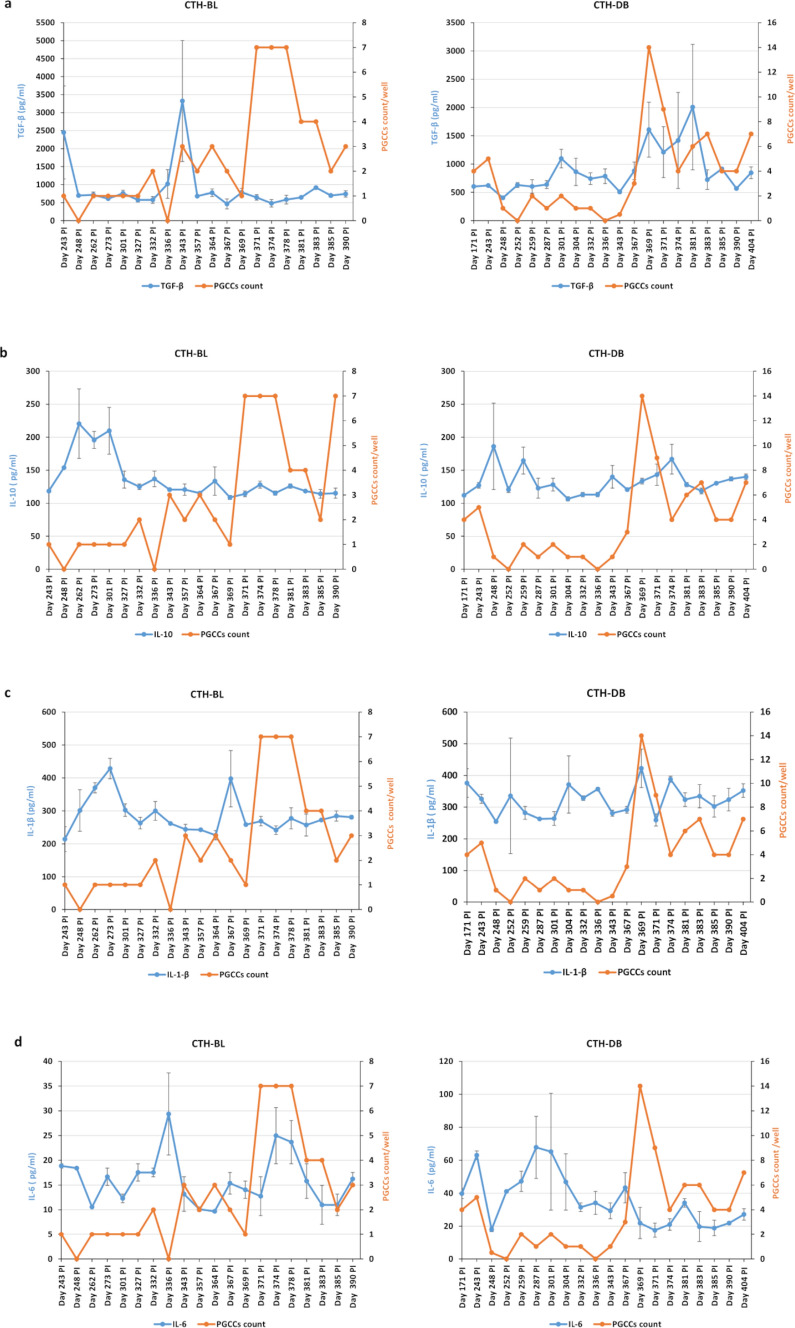
Cytokines production in CTH cultures. Cytokines TGF-β (**a**), IL-10 (**b**), IL-1β (**c**), and IL-6 (**d**) were measured in supernatants of CTH cultures by ELISA kits. Remarkably, TGF-β concentration was enhanced along with the PGCCs count in the CTH-DB culture. IL-10 concentration was reduced in the CTH-BL culture with the presence of a high PGCCs count. The higher IL-6 expression was detected in CTH-DB culture. Data are represented as mean ± SD of two independent experiments. Blue and orange curves represent the cytokine concentration (pg/ml) (Left Y-axis) and PGCCs count/well (Right Y-axis), respectively. The X-axis represents the days post-infection

We assessed cytokines production in CTH cultures using ELISA assays (Fig. 2). The cytokine TGF-β was detected in CTH-BL and DB cultures at a concentration ranging between 500 and 3400 pg/ml. The highest TGF-β concentration was noticed at day 343 post-infection in CTH-BL culture. Remarkably, the TGF-β concentration was increased along with the PGCCs count in the CTH-DB culture (Fig. 2a). The production of IL-10 and IL-1β slightly varied between CTH-BL and CTH-DB cultures (Fig. 2b and c). Particularly, IL-10 concentration was decreased in the CTH-BL culture with the presence of a high PGCCs count (Fig. 2b). Higher expression of IL-6 was induced in CTH-DB compared to CTH-BL (Fig. 2d).

### Correlation between PGCCs/cytokine production depends on the HCMV strain (DB versus BL)

**Fig. 3:**
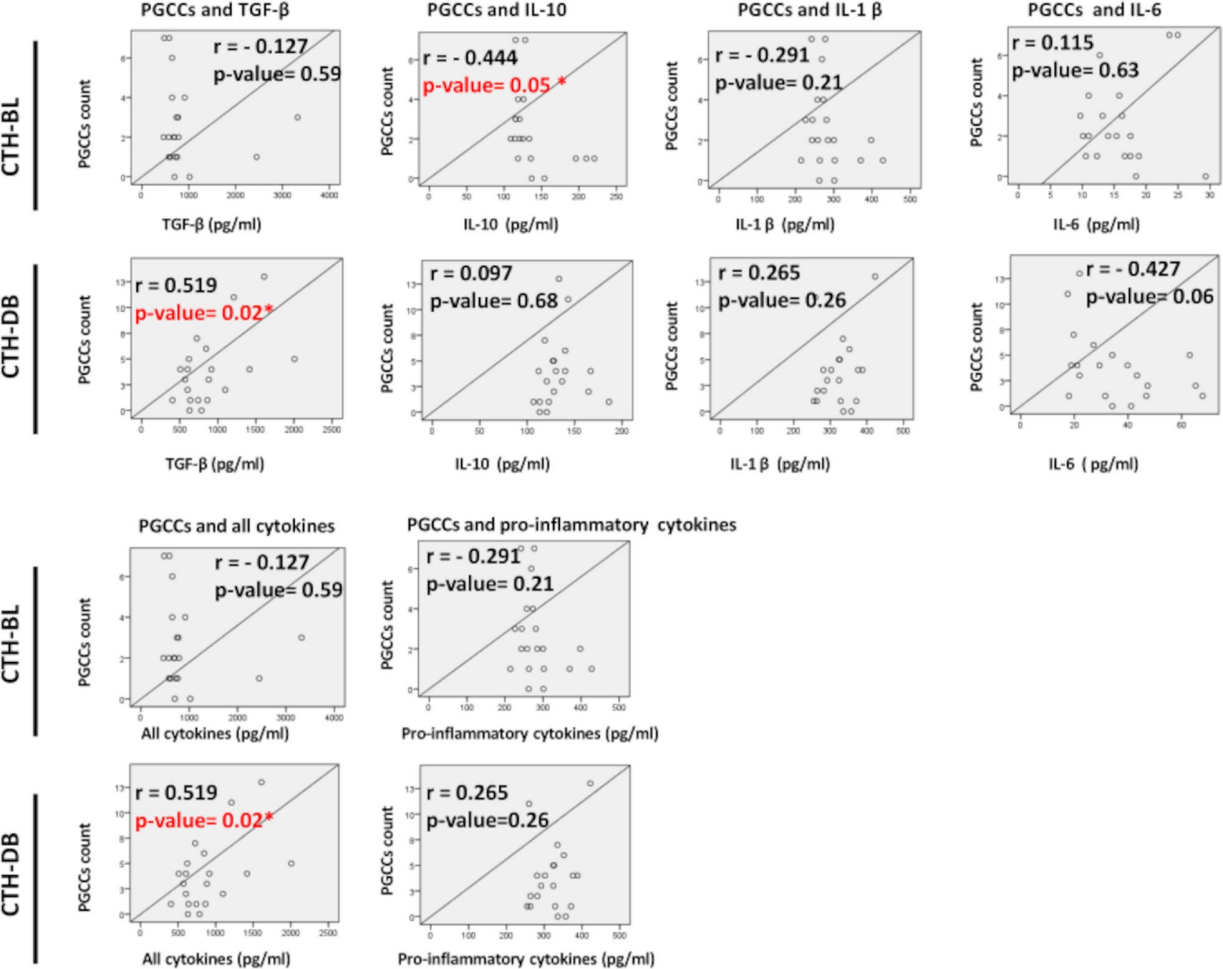
Correlation between PGCCs count and cytokines production in CTH cultures. Pearson’s correlation between PGCCs count and cytokine production was conducted in CTH cultures. A statistically significant negative correlation was detected between IL-10 expression and PGCCs count in CTH-BL culture. Moreover, a significant strong positive correlation was observed between PGCCs count and TGF-β or all cytokines production in CTH-DB cultures. The X-axis represents cytokine concentration (pg/ml), and the Y-axis represents PGCCs count. All cytokines: TGF-β, IL-6, IL10, IL-1β, Pro-inflammatory cytokines: IL-1β and IL-6. (r) stands for correlation coefficient and (*) shows significant p-value ≤ 0.05

**Fig. 4:**
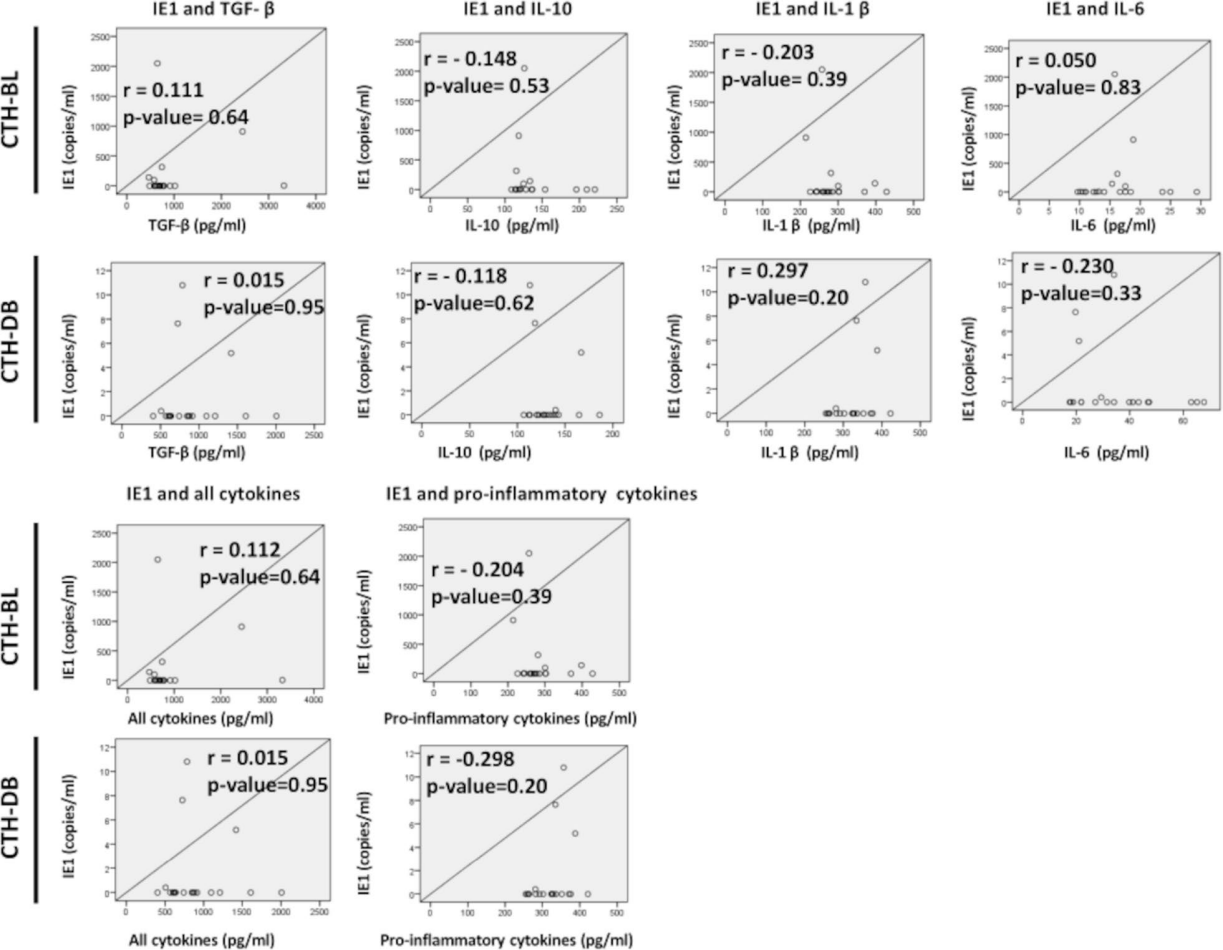
Correlation between HCMV presence and cytokines production in CTH cultures. Pearson’s correlation between viral load and cytokine production was conducted in CTH cultures. No significant correlation was identified between IE1 gene expression and cytokines production. The X-axis represents cytokine concentration (pg/ml), and the Y-axis represents viral load as measured by IE1 detection using qPCR (copies/ml). All cytokines: TGF-β, IL-6, IL10, IL-1β, Pro-inflammatory cytokines: IL-1β and IL-6. (r) stands for correlation coefficient

We analyzed the link between viral load or PGCCs presence and cytokines production using Pearson’s correlation test. We noticed a statistically significant negative correlation between IL-10 expression and PGCCs count in CTH-BL (r = -0.444, p-value = 0.05) (Fig. 3 and Table 1). Furthermore, TGF-β or all cytokines production were strongly and positively correlated with PGCCs count in CTH-DB culture (r = 0.519, p-value = 0.02, and r = 0.519, p-value = 0.02, respectively) (Fig. 3). No other statistically significant correlation was detected between the remaining cytokines and PGCCs count (Table 1. For example, a non-significant negative correlation was detected between PGCCs count and IL-6 production in CTH-DB culture (r = -0.427, p-value = 0.06) (Fig. 3). No significant correlation was found between HCMV presence (IE1 gene) and cytokines production (Fig. 4).

**Table 1:** Correlation between PGCCs count and cytokines production in CTH cultures

	Correlation between PGCCs and the cytokines below	r	p-value
CTH-BL	TGF-β	− 0.127	0.59
CTH-DB		0.519	0.02*
CTH-BL	IL-10	− 0.444	0.05*
CTH-DB		0.097	0.68
CTH-BL	IL-1β	− 0.291	0.21
CTH-DB		0.265	0.26
CTH-BL	IL-6	0.115	0.63
CTH-DB		− 0.427	0.06
CTH-BL	All cytokines	− 0.127	0.59
CTH-DB		0.519	0.02*
CTH-BL	Pro-inflammatory cytokines	− 0.291	0.21
CTH-DB		0.265	0.26

All cytokines: TGF-β, IL-6, IL10, IL-1β, Pro-inflammatory cytokines: IL-1β and IL-6. (r) stands for correlation coefficient and (*) shows significant p-value ≤ 0.05. *PGCCs* Polyploid giant cancer cells, *CTH* Cytomegalovirus-Transformed HMECs, *TGF-β* Transforming growth factor beta, *IL* Interleukin

### Detection of PGCCs and cytokines, as well as the correlation between PGCCs/cytokine expression in BC biopsies

**Fig. 5:**
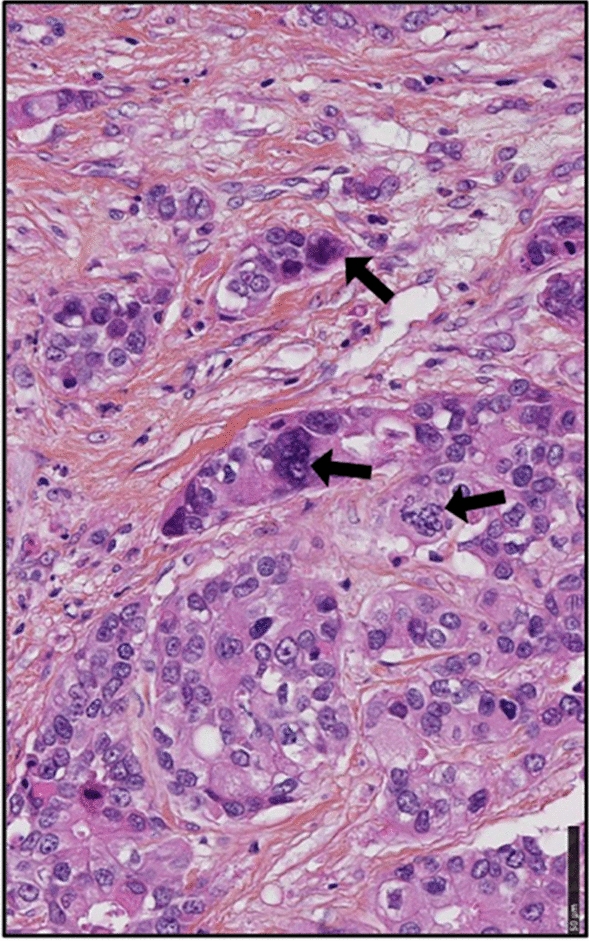
PGCCs detection in breast cancer biopsies. Presence of PGCCs in breast cancer biopsies (arrows). The tissue was stained using HES. Magnification: 40X. Scale bars are 50 μm

**Fig. 6:**
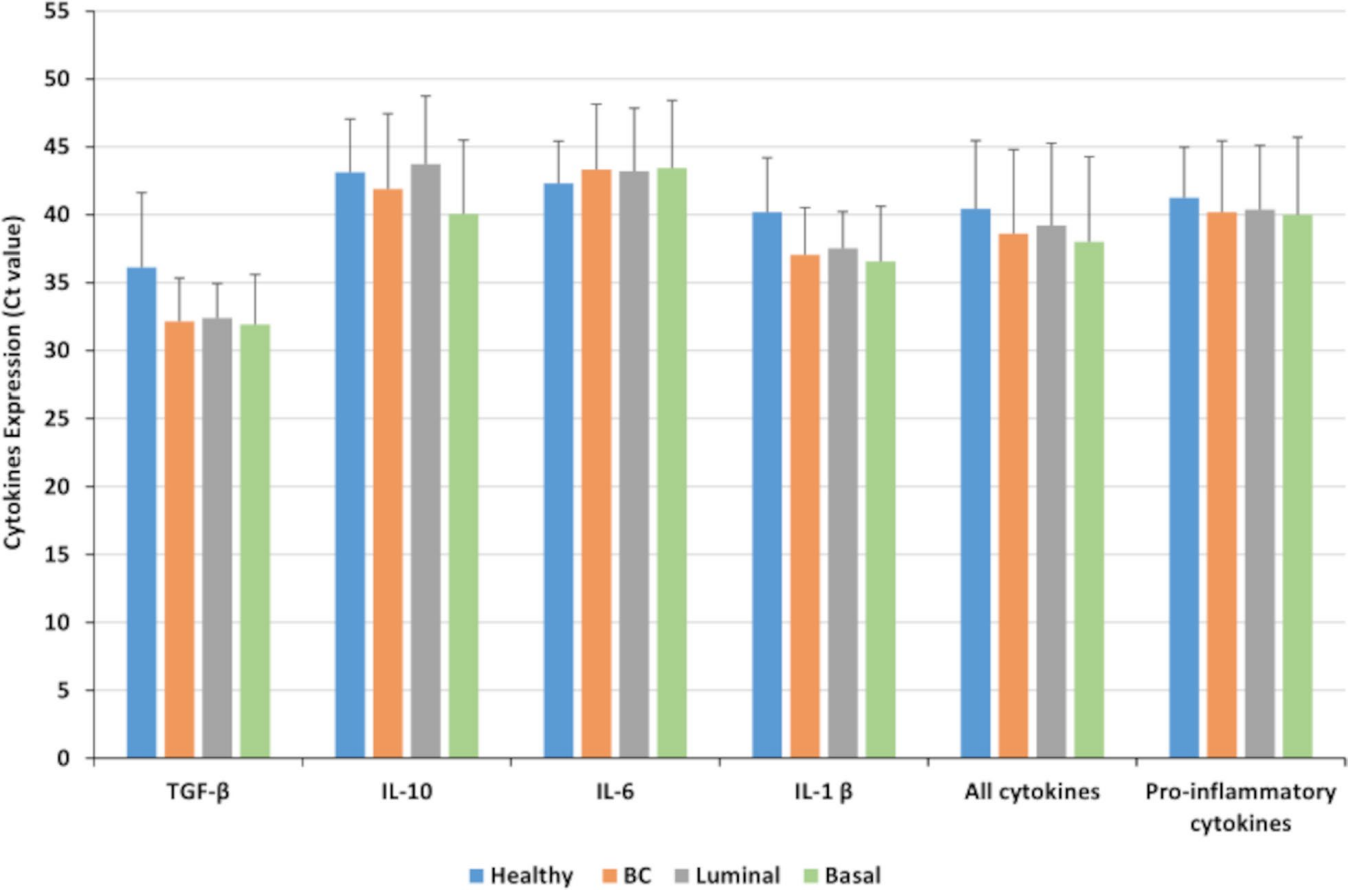
Cytokines expression in breast biopsies. Histogram representing the expression of TGF-β, IL-10, IL-6, IL-1β, all cytokines and pro-inflammatory cytokines in healthy breast biopsies, all breast cancer biopsies (BC), as well as luminal and basal biopsies. Cytokines expression in biopsies was assessed by reverse transcription-quantitative polymerase chain reaction (RT-qPCR). Data are represented as mean ± SD of two independent experiments

**Fig. 7:**
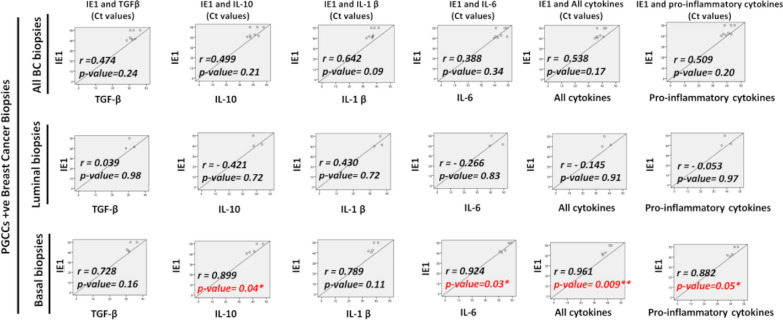
Correlation between HCMV presence and cytokines expression in PGCCs-positive breast biopsies. Pearson’s correlation between HCMV load and cytokines expression was conducted in luminal and basal breast cancer biopsies. A statistically significant strong positive correlation was observed between HCMV presence and IL-10, IL-6, all cytokines, and pro-inflammatory cytokines in PGCCs-positive basal-like biopsies. The X-axis represents cytokine expression (Ct value), and the Y -axis represents viral load as measured by IE1 qPCR (Ct value). All cytokines: TGF-β, IL-6, IL10, IL-1β, Pro-inflammatory cytokines: IL-1β and IL-6. (r) stands for correlation coefficient. *p-value ≤ 0.05; **p-value ≤ 0.01

To further decipher the relation between PGCCs count and cytokine production in vivo, we analyzed sixteen breast cancer biopsies (luminal n = 8 and basal-like n = 8) parallel to four healthy mammary biopsies for the presence of PGCCs, HCMV, as well as cytokine expression. The pathological data for the sixteen breast cancer biopsies were provided in Table 2. PGCCs with giant or multiple nuclei were detected in human breast cancer biopsies, in particular basal-like breast cancer biopsies (Fig. 5, arrows). Compared to healthy biopsies, high expression of TGF-β, IL-1β, and IL-10 was reported in BC biopsies, notably in basal-like biopsies; a slight variation in IL-6 expression was noticed in BC biopsies (Fig. 6). Hence, all cytokines as well as pro-inflammatory cytokines were highly expressed in basal-like BC biopsies compared to healthy biopsies (Fig. 6). As we previously confirmed the positive correlation between PGCCs count and HCMV presence in basal-like breast cancer, we assessed the expression of cytokines in these PGCCs-positive basal-like biopsies. A statistically significant strong positive correlation was identified between HCMV presence and IL-10, IL-6, all cytokines, and pro-inflammatory cytokines in PGCCs-positive basal-like biopsies (r = 0.899 p-value = 0.04, and r = 0.924, p-value = 0.03, r = 0.961, p-value = 0.009, and r = 0.882, p-value = 0.05, respectively) (Fig. 7).

**Table 2:** Pathological data of breast cancer biopsies

	Biopsies #	ER	PR	HER2	Histo Type	Elston Ellis Grade	Vascular emboli	TNM
Luminal biopsies	#1	95	10	0	Lobular	II (3,2,1)	No	T2N0Mx
	#2	100	95	0	Ductal	II (2,2,2)	No	T2N0Mx
	#3	95	90	0	Ductal	II (3,2,1)	No	T1cN0Mx
	#4	95	70	0	Ductal	II (3,2,2)	No	T2N1Mx
	#5	100	100	0	Ductal	I (2,1,1)	No	ND
	#6	80	90	0	Lobular	II (3,2,1)	No	T2N1Mx
	#7	95	95	0	Ductal	I (2,2,1)	No	T1cN1mi
	#8	99	15	0	Ductal	III (3,2,3)	No	T3N1miMx
Basal biopsies	#9	0	0	0	Ductal	III (3,3,3)	No	T2N2aMx
	#10	0	0	0	Ductal	III (3,2,3)	ND	ND
	#11	0	0	0	Ductal	III (3,3,3)	Yes	ND
	#12	0	0	0	Ductal	III (3,3,3)	Yes	ND
	#13	0	0	0	Ductal	III (3,3,3)	Yes	ND
	#14	0	0	0	Ductal	III (3,3,2)	Yes	ND
	#15	0	0	0	Ductal	III (3,3,3)	Yes	T2N1mi
	#16	0	0	0	Ductal	III (3,2,3)	ND	T2N0

*ND* Not determined

## Discussion

Polyploid giant cancer cells (PGCCs) were previously found in vitro and in vivo, and are especially noticeable in poorly differentiated, late-stage, and treatment-resistant cancers [44–46]. PGCCs count is increased in high breast tumor grade and lymph node metastases which suggests a relation between PGCCs and tumor recurrence potential [3]. Moreover, PGCCs were found in MDA-MB-231 and MCF7 breast cancer cell lines [43, 47, 48]. PGCCs induced a mesenchymal phenotype and displayed stem-like properties [3, 43, 46]. In line with these studies, PGCCs were mainly detected in basal-like compared to luminal biopsies. Further, our CTH cells that exhibit PGCCs following chronic HCMV infection were described to display mesenchymal and embryonic-like stemness features [17, 18]. As oncoviruses might trigger PGCCs formation [5], it is worth mentioning the absence of any other oncovirus in our viral stocks and CTH culture, thus confirming that the detected PGCCs phenotype is due to HCMV.

Furthermore, cytokines were described to regulate the induction and protection in breast cancer [31]; overexpression of several cytokines was described in estrogen receptor-negative breast carcinoma [26]. We mainly detected high expression of TGF-β, IL-1β, and IL-10 in basal-like breast cancer compared to healthy and luminal biopsies. In agreement with our data, TGF-β and IL-6 were described to promote epithelial to mesenchymal transition by downregulating the expression of cell adhesion genes and upregulating the cell motility genes and genes associated with the mesenchymal phenotype [24, 32–34, 49]. This regulation could lead to a complete mesenchymal process (C-EMT) which was strongly associated with basal-like tumors [50, 51]. Additionally, ER-positive breast tumor cells were described to produce lower levels of IL-6 than ER-negative breast tumor cells [52]. Overexpression of IL-6 exhibits an epithelial-to-mesenchymal transition (EMT) phenotype in MCF7 cells and promotes their invasiveness [53]. In line with this study, higher IL-6 production was detected in CTH-DB cultures that previously exhibited more EMT features compared to CTH-BL cultures [17, 18]. However, the inhibition of IL-6 and IL-8 in TNBC cell lines decreased cell survival as well as colony formation, and prevented tumor growth in vivo [54]. Likewise, the expression of IL-1β was mainly reported in a highly malignant invasive mammary cell line (such as MDA-MB-231) [28, 55, 56]. In agreement with our in vivo outcomes, high levels of IL1-β were linked to breast cancer aggressiveness and poor prognosis [26, 57]. In addition, high expression of IL-10 was reported in breast tumors [26, 58].

Cytokines play a critical role not only in tumor growth and metastases but also in tumor evasion.

For instance, the anti-inflammatory cytokine IL-10 inhibits cytokine production and antigen presentation by T cells and macrophages [38, 39]. TGF-β was described to suppress natural killer cells (NK) and promote regulatory T-cell activity through a neuropilin-1-mediated mechanism [24, 59, 60]. This could explain the high IL-10 and TGF-β expression detected in the poor prognosis basal-like breast cancer biopsies compared to the luminal and healthy breast biopsies. Additionally, HCMV-infected tumor cells produce immunosuppressive cytokines such as IL-10 to escape the immune responses and counteract the pro-inflammatory cytokines production [8, 61]. This might be in line with the significant strong correlation detected between HCMV replication (IE1 expression) and IL-10 production in PGCCs-positive basal-like biopsies.

The HCMV-encoded IL-10, a homolog of the potent human interleukin 10 (hIL-10), possesses a range of immunomodulatory functions, including suppression of pro-inflammatory cytokine production. [62–64]. During viral latency, the expression of latency-associated cmvIL-10 (LAcmvIL-10), another isoform of the virus-encoded IL-10, modulates the microenvironment of infected cells and allows immunity evasion [65]. Furthermore, the immune suppressive cytokine TGF-β was reported to stimulate HCMV replication in fibroblast cultures [66]. HCMV produces TGF-β in different tumor cell types including glioblastoma, leukemia, and osteosarcoma cells [67, 68]. The HCMV IE2 protein enhances TGF-β gene transcription by interacting with the Egr-1 DNA-binding protein [69]. Moreover, a comparative study showed that HCMV-IE proteins activated the TGF-β promoter in the absence and presence of HCMV infection [68]. Hence, the production of TGF-β by HCMV could influence the infected cells, neighboring tissues and immune responses to benefit from the stimulating viral replication and avoid immune responses through the negative regulatory effects of TGF-β on lymphocytes functions [9, 70, 71]. This could further explain the enhanced expression of TGF-β detected in CTH cultures and basal-like biopsies.

Latency was reported to be essential for transformation induced by oncogenic herpesviruses [17, 72]. HCMV persistence was described in tubular epithelial cells [73], neural stem cells [74], and osteogenic sarcoma-derived cells [75]. Furthermore, the detection of some lytic blips suggests that both lytic viral replication and viral latency are essential to promote and maintain CTH transformation as previously reported for the other two oncogenic herpesviruses, namely EBV and Kaposi's sarcoma-associated herpesvirus (KSHV) [76, 77]. In addition, the detection of different PGCCs count and cytokine concentrations in CTH cultures suggests that the diversity of HCMV strains could affect the exhibition of PGCCs and cytokines production. Several studies showed that HCMV disease and pathogenesis could be related to HCMV genome diversity [78, 79]. Increasing evidence suggests an association between HCMV genetic diversity and HCMV pathogenesis that could also modulate oncomodulation/oncogenesis [80, 81].

PGCCs detection and cytokines expression depend not only on breast cancer type but also on the tumor microenvironment (TME). The composition of TME is modified by PGCCs through the recruitment of diploid cancer cells from adjacent zones; these diploid cells can ultimately become PGCCs [82]. Additionally, PGCCs progeny formation was described to be a part of the TME. PGCCs stemness, metastasis, vasculogenic mimicry and chemoresistance were recognized as the outcomes of the dynamic relationship between PGCCs and the TME [1]. Tumor cells secrete cytokines that recruit and activate other cells in the TME. Moreover, cytokines induce a tumor-supportive immune microenvironment by inhibiting anti-tumor immunity [83]. The inactivation of NF-kB in myeloid cells was described to reduce cytokines expression and tumor size [84]. The inflammatory cytokines including IL-1β, IL-6, IL-8, and CCL-5 that are induced by NF-kB promote tumor growth through the induction of cell proliferation [85]. The tumor-associated macrophage (TAMs), tumor-associated neutrophils (TANs), myeloid-derived suppressor cells (MDSC), and regulatory cells (Tregs) present in the TME are associated with poor prognosis. They produce IL-10 and TGF-β that suppress the activity of NK cells, T and B lymphocytes in the TME, allowing the proliferation and survival of cancer cells [85]. Also, TAMs, endothelial cells, and fibroblasts in the TME induce angiogenesis via IL-6, IL-8, and TGF-β [86–88]. TAMs promote metastasis and invasion in breast cancer through the secretion of IL-1β [31, 89]. TAMs, the most abundant cells in the TME, were associated with poor prognosis, especially in basal-like breast cancer [90, 91]. Furthermore, the pro-metastatic microenvironmental factor S100A4 was described to stimulate basal-like breast cancer cells to secrete pro-inflammatory cytokines that convert monocytes into TAM-like cells [92]. In line with this data, we detected the highest expression of the pro-inflammatory cytokines, IL-1β and IL-6, in basal-like breast cancer. Moreover, the abundant presence of TAMs in basal-like breast cancer could explain the high expression of TGF-β and IL-10 detected in basal biopsies compared to luminal ones. Hence, we highlighted the critical relationship between PGCCs/cytokines present in the TME and the basal-like breast cancer. Furthermore, the significant strong correlation identified between HCMV presence and the expression of IL-10, IL-6, all cytokines and pro-inflammatory cytokines suggested that HCMV presence could regulate cytokines expression in basal-like BC biopsies. High HCMV load might favor EMT features and immune evasion in basal-like BC biopsies by upregulating the expression of cytokines, particularly IL-10 and IL-6. Thus, our outcomes underline the strong link between HCMV load, PGCCs presence and cytokines expression in basal-like breast cancer that displays the most aggressive phenotype.

The in vitro PGCCs/cytokine profile present in our CTH model matched the in vivo PGCCs/cytokine profile identified in breast cancer biopsies. The highest IL-6 expression usually linked to EMT and PGCCs count was identified in CTH-DB culture and basal-like biopsies that harbor the most malignant phenotype [17, 18, 93]. Finally, telomere dysfunction-driven polyploidization is a universal source of tumor evolution that occurs continuously during neoplastic cell growth [94]. Oncoviruses deregulate telomerase activity and telomere length and promote cancer development [95]. Interestingly, HCMV activates telomerase [96], and favors the appearance of PGCCs which are considered as hallmarks of oncoviruses [5]. Further studies are needed to investigate the relationship and underlying mechanisms between polyploid cancer cells, cytokines production, and cytomegalovirus.

Our study has some limitations to address. To start with, the limited sample size; higher sample size enhances the significance level of our findings. Furthermore, the restricted cytokine evaluation; assessing more cytokines in CTH cultures and breast cancer biopsies might highlight the potential role of HCMV in cytokine production and cancer progression. Additionally, the absence of characterization of inflammatory cells that might underline the link between HCMV, cytokine production and cancer progression.

## Conclusion 

In conclusion, our findings revealed for the first time an association between high-risk HCMV strains, PGCCs formation, and cytokines production in vitro and in vivo. Our study presents a proof-of-concept for highlighting the cytokine profile in breast cancer, particularly the basal-like breast cancer, parallel to the presence of PGCCs and HCMV detection, thereby opening the door toward new therapeutic approaches in breast cancer patients with poor prognostic characteristics.

## Supplementary Information

**Additional file 1: Table S1**. List of primers used.

## Data Availability

The data supporting the findings of this study are available within the article and its Supplementary Information files and from the corresponding authors on request.
