# Supplementary material for: Polyploid giant cancer cells, cytokines and cytomegalovirus in breast cancer progression
Source: Cancer Cell Int. 2023 Jun 20;23:119. doi: 10.1186/s12935-023-02971-1 (PMC10280854; doi:10.1186/s12935-023-02971-1)
Supplement: Supplementary file 1 — Additional file 1: Table S1. List of primers used. [file 12935_2023_2971_MOESM1_ESM.pdf]

Additional file 1

Table S1: List of primers used.

Gene Name	FWD sequence	Reverse sequence
**TGF-β**	5'-TACCTGAACCCGTGTTGCTCTC-3'	5'-GTTGCTGAGGTATCGCCAGGAA-3'
**IL-10**	5'-TCTCCGAGATGCCTTCAGCAGA-3'	5'-TCAGACAAGGCTTGGCAACCCA-3'
**IL-1β**	5'-AGGCACAAGGCACAACAGGCT-3'	5'-AACAACTGACGCGGCCTGCC-3'
**IL-6**	5'-CATTCTGCCCTCGAGCCCACC-3'	5'-GGCAGCAGGCAACACCAGGA-3'
**GAPDH**	5’-CCCCTCTTCAAGGCCTCTAC-3’	5’-CGACCACTTTGTCAAGCTCA-3’
